# The perspective on standardisation and harmonisation: the viewpoint of the EASI president

**DOI:** 10.1186/s13317-020-0127-3

**Published:** 2020-02-06

**Authors:** Jan Damoiseaux

**Affiliations:** Central Diagnostic Laboratory, Maastricht University Medical Center, P. Debyelaan 25, 6229 HX Maastricht, The Netherlands

**Keywords:** Autoantibody, Harmonisation, Standardisation

## Abstract

Standardisation of immuno-assays for autoantibodies is a major challenge. Although multiple organisations participate in the generation of internationally accepted standards, adequate standardisation of assays has not yet been achieved. Harmonisation may offer an alternative approach to better align requesting, testing, reporting and interpretation of autoimmune diagnostics. The European Autoimmunity Standardisation Initiative (EASI) was founded to facilitate both standardisation as well as harmonisation of autoantibody tests, but over the years the focus has drifted away from standardisation in favour of harmonisation. In the current paper the options for harmonisation are highlighted.

## Introduction

The European Autoimmunity Standardisation Initiative (EASI) was founded in 2006 to stimulate standardisation and harmonisation of autoantibody tests for optimal patient care [1]. Standardisation can be defined as the process of implementing a standard preparation in order to maximize compatibility of test results, eventually resulting in uniformity. Harmonisation, on the other hand, can be defined as the adjustment of differences and/or inconsistencies among different measurements, methods, and procedures to make them uniform or mutually compatible. Harmonisation is typically achieved by agreement as consolidated in recommendations and/or guidelines. Although standardisation has been achieved for multiple laboratory parameters in clinical chemistry and hematology, standardisation of autoantibody assays has appeared a major challenge. Considering that the measurand, i.e., antibodies, consists of a highly variable mixture of molecules that are different in terms of epitope recognition, degree and type of glycosylation, isotype and subclass distribution, and avidity, the awareness has increased that standardisation of autoantibody assays might be an utopia. This is elegantly illustrated in the example of anti-dsDNA antibodies [2]. In the current paper, as president of the EASI Forum Group, I will highlight my personal view on the challenges of autoantibody standardisation and the options of harmonisation in autoimmune diagnostics.

## Standardisation

In the past, several internationally accepted standard preparations for autoantibody detection have been launched by a multitude of distinct organisations [3]. For instance, the World Health Organisation (WHO) prepared standards for rheumatoid factor (RF; W1066 assigned 25 international units (IU)), and anti-dsDNA antibodies (W0/80 assigned 200 IU [4, 5]. The W1066 standard, originally referred to as 64/1, was prepared by the Dutch Bloodbank (Sanquin, Amsterdam) as a serumpool of 197 patients with rheumatoid arthritis (RA). The W0/80 standard, on the other hand, was plasmapheresis material of a single patient with systemic lupus erythematosus (SLE). Also the Autoantibody Standardizing Committee (ASC), a subcommittee of the International Union of Immunological Societies (IUIS) quality assessment and standardization committee has generated a broad panel of reference materials for autoantibody detection, including standards for myeloperoxidase (MPO) anti-neutrophil cytoplasmic antibodies (ANCA) and proteinase 3 (PR3)-ANCA [6]. Both standards were each prepared from plasmapheresis material of single patients with ANCA-associated vasculitis (AAV) and were assigned a value of 100 IU. Although the assignment of IU is a privilege of the WHO, it should be acknowledged that the ASC operates on behalf of the WHO. More recently, standards for MPO-ANCA (ERM-DA476/IFCC) and PR3-ANCA (ERM-DA483/IFCC) were also prepared by the Institute for Reference Materials and Methods (IRMM), in collaboration with the Working Group Harmonisation of Autoantibody Tests (WG-HAT) of the International Federation of Clinical Chemistry and Laboratory Medicine (IFCC) [7, 8]. Also these standards were prepared from plasmapheresis material of single patients with AAV and are assigned a value in mass units. The advantage of the IRMM standards has been claimed to be the commutability, i.e., the equivalence of the mathematical relationships between the results of different measurement procedures for a reference material and for representative samples from healthy and diseased individuals.

**Fig. 1:**
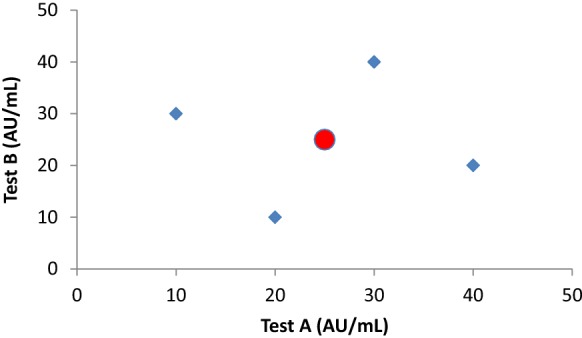
The effect of pool-sera in relation to individual test results. If equal amounts of 4 samples with different reactivities (10, 20, 30 and 40 AU/mL; blue diamonds) in 2 immuno-assays are mixed, the pool-serum probably will reveal the average reactivity (25 AU/mL; red circle) in both assays. If both assays would be calibrated on this pool-serum, the individual test results of the 4 samples will remain different. This obviously also holds for megapools

The main question about the currently available standards for autoantibody diagnostics is what these standards have brought us until today. Evidently, this is not the intended standardisation of test results. In case of AAV the ASC MPO- and PR3-ANCA standards have been used by several diagnostic companies, but this has not resulted in alignment of results [9]. Whether the claimed commutability of the IRMM ANCA standards will solve the problem, remains to be established. The fact that the ASC and IRMM ANCA standards reveal quite similar results within the same immunoassay, but clearly differ from one assay to the other, does not hold great promise for the new standards (Bossuyt et al., manuscript in preparation). The WHO standard for anti-dsDNA antibodies has uncovered another important caveat of standards that have been prepared from a single patient. The stock of the WHO standard has run out and, next, it appeared impossible to replace by a novel standard with the same characteristics. The novel material (15/174), therefore, is not released as a new WHO standard, but only as reference material [10]. Consequently, the reference material has been assigned a nominal value of 100 U/ampoule and, as such, is not defined in IU. Obviously, the problem of not being able to replace a standard preparation, could potentially be solved by making a large pool of serum obtained from multiple patients. Considering the complexity of the idiotype – anti-idiotype network it can be imagined that the autoantibody reactivity changes considerably after pooling the sera. To circumvent this problem, a novel megapool strategy has been applied in the establishment of an international autoantibody reference standard for human anti-DFS70 antibodies [11]. This strategy is based on stepwise pooling of sera and consistently checking for antibody reactivity in several distinct methods after each step of pooling. This reference standard is now integrated in the panel of ASC standards. Although this material is referred to as ‘standard’, the intentional use is for proper assay validation and interpretation of the dense fine speckled HEp-2 indirect immunofluorescent assay (IIFA) pattern (AC-2), i.e., not for standardisation of distinct immunoassays enabling detection of anti-DFS70 antibodies. Nevertheless, it is to be expected that pooling of sera will create a mixture with a broad spectrum of epitopes recognized, glycosylation, and avidities, in combination with an average distribution of isotypes and subclasses. The megapool approach can be compared with the production of intravenous immunoglobulin preparations. By starting with a huge number of donors a wide spectrum of potential antibody variants will be included and lot-to-lot variation will be minimized. As shown for RF by Jacobs and Bossuyt, such pools become more commutable, resulting in similar results for the pool of sera in different immunoassays [12]. However, even standards based on pooled sera, such as the W1066 RF standard, may give aberrant results in distinct immunoassays that have been calibrated on this standard and express the results in IU [13]. Similar conclusions were derived from a Dutch study using an alternative pool of RF sera as reference [14], the so-called Reference Laboratory for Rheumatologic Serology (RELARES) with a defined IgM-RF level of 200 IU/mL [15]. Not surprisingly, individual sera still revealed quite different results in these immunoassays (Fig. 1 and [12]). Apparently, the source of the autoantigen and the way the autoantigen is presented in the immunoassay are critical parameters for taking into account if standardisation is to be achieved.

## Harmonisation

**Fig. 2:**
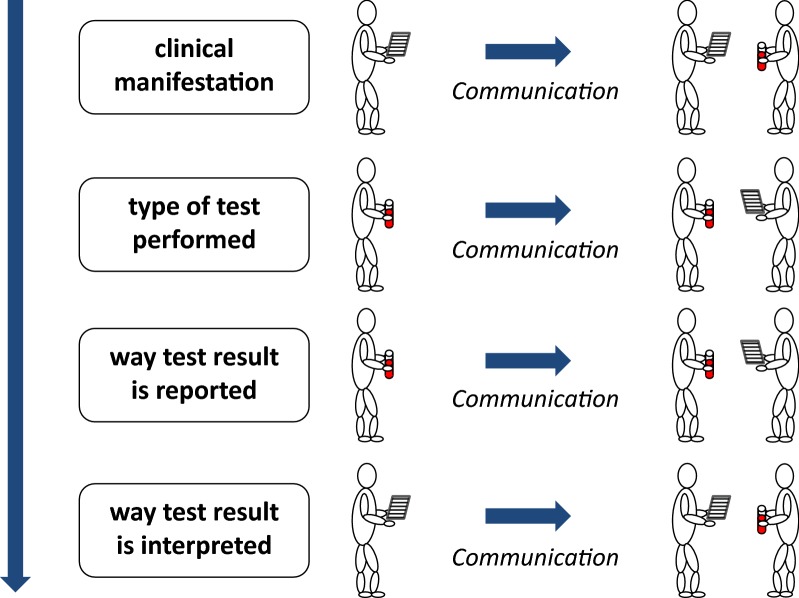
The patient-oriented added-value of a test result

Because of the difficulties encountered in the process of standardisation, EASI has shifted the focus from standardisation toward harmonisation. As precipitated in the definition of harmonisation, this is to be achieved by recommendations and guidelines. This can happen at multiple levels (Fig. 2) and typically requires optimal bidirectional communication between the clinician and the laboratory specialist [16].

The first level of harmonisation involves the definition of clinical manifestations that warrant the request for a certain autoantibody test. Due to automation of autoantibody serology many assays, nowadays, are widely available, even outside laboratories specialised in autoimmune diagnostics. This has enormously increased the number of requests and diversified the clinical disciplines requesting such assays at the cost of a valid pre-test probability as defined by the clinical manifestations of the patient. This is best exemplified by the situation for anti-nuclear antibodies (ANA), eventually resulting in positive results that are not easily explained by the clinical manifestations being apparent at the time of presentation [17]. In case of AAV, the first international consensus on ANCA testing clearly defined the clinical manifestations that justify the request for an ANCA test [18]. This gating strategy has been proven to be effective in several studies [19–21], and therefore was reinforced in the 2017 international consensus on ANCA testing [22]. Obviously, in most diagnostic and/or classification criteria the defined clinical criteria implicitly include the clinical manifestations that call for autoantibody diagnostics [23–25].

Based on the clinical manifestations, to be communicated by the clinician to the laboratory specialist, the optimal testing conditions can be applied in the laboratory. In particular for the systemic autoimmune diseases the autoantibody testing is precipitated in an algorithm. For the ANA-associated rheumatic diseases (AARD), the HEp-2 IIFA has been advocated as the most optimal screening test [26, 27], but this is currently being challenged because this might be different for the distinct AARD entities [28, 29]. In case of the idiopathic inflammatory myopathies (IIM), screening with HEp-2 IIFA may not be the best choice, although the currently available multiplex immunoassays for IIM-specific autoantibodies also harbour some challenges [30, 31]. Importantly, reference to assay specification, or the lack thereof, in classification criteria for these diseases also may impact the testing algorithm. For instance, the classification criteria for systemic sclerosis (SSc) include anti-centromere antibodies in the domain of relevant autoantibodies [24]. For these anti-centromere antibodies it is sufficient to identify the centromere pattern in the HEp-2 IIFA. The new ACR/EULAR criteria for SLE allow the use of alternative immunoassays instead of the HEp-2 IIFA if such an assay at least has an equivalent performance [25]. Since ANA are taken as an entry criterium, this equivalent performance should primarily be based on high sensitivity, but, unfortunately, the definition of equivalent performance is not provided. Whatever the choice of screening test, a positive test is intended to be followed by antigen-specific immunoassays in order to identify autoantibodies more specific for a certain disease type. In general, these follow-up tests for AARD enable detection of about 6–10 distinct autoantibodies. The availability of clinical information, as far as appropriate, will enable to select for disease-specific profiles as available for IIM and SSc. Since in several countries automatic reflex testing is not allowed, the laboratory specialist at least can advise the clinician to request for analysis of the most relevant autoantibodies. While the discussion on the most optimal screening test for AARD is still on-going, for AAV it has been shown in a recent multi-center study that screening with antigen-specific immunoassays, i.e., detection of MPO- and PR3-ANCA, is to be preferred above screening by IIFA on ethanol-fixed neutrophils [32], and this finding has resulted in revision of the international consensus on ANCA testing for AAV [22]. The latter is a clear example of harmonisation by an evidence-based consensus on the testing algorithm for an autoimmune disease.

Next, the test-results have to be reported to the clinician, preferentially in a universal manner. It is important that the results are reported in the context of reference values and with information about the methods used. For quantitative assays, in general, two cut-offs are provided that indicate a grey-zone. The highest cut-off is considered the upper limit of normal and this is often referred to in the context of diagnostic and classification criteria, for instance for coeliac disease and RA [23, 33]. Unfortunately, there is substantial difference in the way diagnostic companies define the cut-off of their immuno-assays and this contributes to differences in test characteristics [13, 32]. Harmonization in reporting of test-results could be achieved if results were to be reported in likelihood ratio’s (LR) and, in particular, in LR for test-result intervals [34]. Obviously, also for calculating a single LR or multiple LR for test-result intervals, the cut-off or multiple cut-offs, respectively, are important. This approach has proven to be very effective for distinct ANCA immuno-assays [35]. One step further is to report LR for each quantitative result instead of arbitrary units [36]. An important caveat of reporting in LR might be that LR for the same immuno-assays may be different for distinct geographical areas due to, for instance, variation in ethnicity or infectious burden. World-wide multi-center studies of sufficient magnitude have to be performed in order to validate to what extent LR can be generalized. Another challenge is the universal use of terminology and definitions. In particular in diagnostic and classification criteria for autoimmune diseases the terminology in relation to autoantibodies is poorly defined. In the recent classification criteria for SLE it is not clear if ANA include only antibodies to nuclear antigens, or also to cytoplasmic antigens [25]. There is no consensus on this issue between different countries [37], but this evidently has impact on the test-characteristics. Similarly, the original classification criteria for Sjögren’s syndrome include autoantibodies to SSA, but there is no differentiation between autoantibodies to SS-A/Ro60 and Ro52/TRIM21 [38]. In a later publication about the clinical practice of the Sjögren’s syndrome, it is stated that only anti-SS-A/Ro60 antibodies have to be considered, because isolated anti-Ro52/TRIM21 antibodies are not specific for the Sjögren’s syndrome [39]. Although this latter conclusion is in line with daily life experience, the original publication could not differentiate between both autoantibodies because in the patient cohorts used to define and validate the criteria this distinction was not made [38]. The lack of clear-cut definitions for terminology used in autoimmune diagnostics is also reflected in routine clinical practice and this may result in misinterpretation of test-results by the clinician. In particular in the field of the HEp-2 IIFA, the International Consensus on ANA Patterns (ICAP) has made important advances in harmonizing the terminology [40]. ICAP has reached consensus on the name and definition of multiple HEp-2 IIFA patterns. Moreover, since the ICAP information has been translated in multiple languages, implementation of ICAP is strongly facilitated.

Finally, the clinician has to interpret the reported test result in the context of the clinical manifestations of the patients. Evidently, reporting in LR would facilitate correct test-result interpretation, especially if the relation between pre- and post-test probability is graphically presented as a function of the LR [34]. Such graphics could also give insight in the way test-results for autoantibodies are integrated in diagnostic and classification criteria. While in the classification criteria for RA both IgM RF and ACPA have been assigned the same value, it is evident that high-level IgM RF have a similar performance as low-level ACPA as defined by the relation between pre- and post-test probability presented as a function of the LR [41]. The challenge for the clinician is to make a good estimation of the pre-test probability based on the (combination of) clinical manifestations. For this, it would be helpful to define the pre-test probability of the clinical manifestations that warrant the request of the test. Although the concept of LR seems to be restricted to quantitative immuno-assays, it is also applicable for HEp-2 IIFA as far as fluorescence intensity values are provided [42]. Alternatively, ICAP has defined the clinical relevance of distinct HEp-2 IIFA patterns in order to support the clinician in requesting adequate follow-up tests in the context of the differential diagnosis [43]. Also for this step in the diagnostic work-up of the patient it is relevant that there is communication between the clinician and the laboratory specialist. Knowing the final diagnosis enables the laboratory specialist to monitor test performance of the respective immuno-assay.

## Conclusions

For standardisation in autoimmune diagnostics to become a reality, multiple hurdles have to be taken (Table 1). Current approaches evidently have not resulted in achieving the goal of standardisation. One of the problems with standards derived from patient material is the limited availability and poor reproducibility after replacement. Restrictions in the variation of distinct aspects of the immunoassays, like the source of the autoantigen, might further help to achieve standardisation, but this is, obviously, hampering the introduction of innovations in autoantibody detection. Altogether, standardisation in autoimmune diagnostics seems to be an utopia.

**Table 1:** Definitions and requirements for standardisation and harmonisation

	Standardisation	Harmonisation
Definition	Implementation of a standard preparation in order to maximize compatibility of test results, eventually resulting in uniformity	Adjustment of differences and/or inconsistencies among different measurements, methods and procedures to make them uniform or mutually compatible
Requirements	Establishment of an internationally accepted measuring unit as defined by a standard preparation	Consensus on clinical manifestations that warrant the request of the test; to be defined in guidelines
	Application of the measuring unit in a wide variety of immuno-assays	Consensus on testing algorithms to be used for distinct autoimmune diseases; to be defined in guidelines
	Implementation of the standardised measuring unit reveals identical test results in individual samples, independent of the immuno-assay of choice	Consensus on reporting of autoantibody results in combination with test characteristics; to be defined in guidelines
	Well-defined composition of the standard preparation in order to guarantee replacement by identical standard preparation	Optimal communication between laboratory specialist and clinician for adequate interpretation of test results

Harmonisation, on the other hand, might be more feasible since this can be achieved by reaching a consensus on defining which clinical manifestations warrant the request of a specific autoantibody test, on the optimal testing algorithm for a specific autoimmune disease, on the way test-results are to be reported to the clinician, and the way these test-results are interpreted in the clinical context of the patient (Table 1). Bidirectional communication between clinician and laboratory specialist is an essential element in this process of harmonisation and this requires clear-cut definitions of the terminology used. Evidently, also for harmonisation there are multiple challenges. Besides lacking data, i.e., potential geographical differences in LR or pre-test probabilities of defined clinical manifestations, reaching consensus on the items mentioned requires close collaboration between the clinical parties involved in autoimmune diagnostics. These parties should also include organisations like the ACR and EULAR that are involved in defining diagnostic and classification criteria, as well as the diagnostic industry. Better defining, and possibly renaming, of terminology is an item that could be initiated by EASI. As for other terms, it will not be easy if EAS(tandardisation)I has to change its name in EAH(armonisation)I.

## Data Availability

Not applicable.
